# Photobiomodulation combination therapy as a new insight in neurological disorders: a comprehensive systematic review

**DOI:** 10.1186/s12883-024-03593-4

**Published:** 2024-03-19

**Authors:** Narmin Farazi, Hanieh Salehi-Pourmehr, Fereshteh Farajdokht, Javad Mahmoudi, Saeed Sadigh-Eteghad

**Affiliations:** 1 Neurosciences Research Center, Tabriz University of Medical Sciences, Tabriz, 5166614756 Iran; 2 Research Center for Evidence-Based Medicine, Iranian EBM Centre: A Joanna Briggs Institute (JBI) Center of Excellence, Tabriz University of Medical Sciences, Tabriz, Iran

**Keywords:** Photobiomodulation, Laser therapy, Combined therapies, Neurological disorders

## Abstract

Preclinical and clinical studies have indicated that combining photobiomodulation (PBM) therapy with other therapeutic approaches may influence the treatment process in a variety of disorders. The purpose of this systematic review was to determine whether PBM-combined therapy provides additional benefits over monotherapies in neurologic and neuropsychiatric disorders. In addition, the review describes the most commonly used methods and PBM parameters in these conjunctional approaches.

To accomplish this, a systematic search was conducted in Google Scholar, PubMed, and Scopus databases through January 2024. 95 potentially eligible articles on PBM-combined treatment strategies for neurological and neuropsychological disorders were identified, including 29 preclinical studies and 66 clinical trials.

According to the findings, seven major categories of studies were identified based on disease type: neuropsychiatric diseases, neurodegenerative diseases, ischemia, nerve injury, pain, paresis, and neuropathy. These studies looked at the effects of laser therapy in combination with other therapies like pharmacotherapies, physical therapies, exercises, stem cells, and experimental materials on neurological disorders in both animal models and humans. The findings suggested that most combination therapies could produce synergistic effects, leading to better outcomes for treating neurologic and psychiatric disorders and relieving symptoms.

These findings indicate that the combination of PBM may be a useful adjunct to conventional and experimental treatments for a variety of neurological and psychological disorders.

**Supplementary Information:**

The online version contains supplementary material available at 10.1186/s12883-024-03593-4.

## Introduction

Neurological disorders cause the majority of disability and are the second leading cause of death worldwide. Over the last three decades, the total number of deaths and disabilities caused by neurological diseases has increased significantly, particularly in low- and middle-income countries [1]. Congenital defects, epigenetic changes, aging, and environmental health issues are the primary causes of the onset and progression of various neurological disorders, which affect both the central and peripheral nervous systems (CNS and PNS) [2–4].

Photobiomodulation (PBM) is a non-invasive physical treatment modality that uses low-level lasers (from the red to near-infrared spectrum, with intensities ranging from 1–500 mW) and/or light-emitting diodes (LEDs) [5]. Evidence suggests that PBM could boost mitochondrial function by improving the electron transfer chain and increasing adenosine triphosphate (ATP) synthesis, as well as lowering oxidative stress biomarkers and inhibiting neuroinflammation [6]. PBM has been used to treat a variety of CNS and PNS disorders, including traumatic brain injury [7], stroke [8], Parkinson’s disease (PD) [9], Alzheimer’s disease (AD) [10], depression, anxiety, cognitive impairments [11, 12], spinal cord injury [13], and carpal tunnel syndrome (CTS) [14]. Both preclinical and clinical studies have shown that PBM therapy improves CNS function [15, 16] and effectively inhibits inflammation in peripheral nerves [17].

Currently, numerous PBM clinics and medical device manufacturers are actively working to improve the parameters that influence PBM effectiveness in the treatment of neurological disorders [18]. The safety of this technique was evaluated in three large randomized clinical trials on acute stroke, known as the "NeuroThera Effectiveness and Safety Trials" (NEST-1, NEST-2, and NEST-3), which found no adverse effects [19–21]. While there have been numerous peer-reviewed articles on PBM, there are few standard RCTs to definitively determine the clinical efficacy of this therapeutic approach [22].

There are some important gaps in the field of PBM therapy that must be addressed. Optimizing neural tissue stimulation with this technique is one of the most difficult challenges, due to the diverse types and severity of neuropathologies, as well as the rapid attenuation of light transmission in tissue [23, 24]. Combination therapies have been proposed as a way to increase treatment efficacy while avoiding severe side effects. As a result, current experimental and clinical studies concentrate on combination therapy rather than single therapy, indicating potential future clinical combination treatment schedules.

Although several systematic reviews have examined the effects of PBM on various neurological disorders [13, 14, 24, 25], we were unable to locate a comprehensive systematic review on PBM combination therapy in neurologic and neuropsychiatric disorders. This review aims to provide an overview of published procedures for determining whether combination therapies for CNS and PNS disorders are more effective than monotherapies. To that end, PBM-based methodologies were tested for detecting and treating neurologic and neuropsychiatric disorders such as depression, anxiety, Alzheimer's disease, Parkinson's disease, stroke, traumatic brain injury, neuropathic pain, spinal cord injury, sciatic nerve crush, paresis, and facial nerve injury. Furthermore, the parameters involved in these procedures were evaluated.

## Methods

### Search strategy

According to the PRISMA (Reporting Items for Systematic Reviews and Meta-Analyses) guidelines, the findings of this review were reported. The Google Scholar, PubMed, and Scopus databases, as international electronic databases, were independently searched up to January 2024 to retrieve all types of studies primarily focused on the synergistic and complementary effects of PBM-combined strategies in the treatment of neurologic and neuropsychiatric disorders. The following keyword combinations based on MeSH terms were used: Photobiomodulation; PBM; Low-Level Laser Therapy; LLLT; Low-Level Light Therapy; Light-Emitting Diode; LED; Combine; Central Nervous System; CNS; Peripheral Nervous System; PNS; Neuropsychiatric; Neurodegenerative; Paresis; Neuropathy; Ischemia; Nerve Injury; pain. The search strategy is presented in Appendix 1. Before selection, duplicate citations were eliminated using the Endnote software. Two independent investigators scrutinized all titles, abstracts, and full texts of potentially qualified articles based on the eligibility criteria, and any discrepancies were resolved by the analysis of a third independent author and the majority consent was taken. Moreover, the reference lists from each article were checked through hand searching to find articles that the search strategy could not have found.

### Study selection and data extraction

Following the search, all English-language published original articles on animal studies and clinical trials were included. The exclusion criteria were in vitro (i.e., cell culture) studies, literature reviews, case reports, protocol studies, conference abstracts, non-English articles, duplicate studies with the same ethical approval number, studies on a combination of two or more lasers at different wavelengths, and acupuncture lasers.

To clarify the relevant details from each included article, data and information from each study were extracted and tabulated as follows: author name and publication year, disease category, species (sex and age), type of combination therapy, laser properties (wavelength, energy density), duration of treatment, and key outcomes. Due to the high variability in the type of disease and treatment meta-analysis was not performed.

### Study quality

The Cochrane Collaboration tool was utilized to evaluate the risk of bias (RoB) in randomized controlled clinical trials. This tool consists of several components including selection bias, performance bias, detection bias, attrition bias, and reporting bias. Animal studies were scored based on a modified version of the CAMARADES’ study quality checklist. The CAMARADES checklist is a reliable and commonly used tool that offers mentoring to those who conduct systematic reviews and meta-analyses of data from preclinical literature [26, 27]. The questions in this tool covers information about publication in a peer-reviewed journal, control of temperature, random allocation to treatment or control, blinded induction, blinding of outcome assessment, use of anesthetic without significant intrinsic neuroprotective activity, animal model, sample-size calculation, compliance with animal welfare regulations, and a statement regarding possible conflicts of interest.

The assessment was conducted by two independent authors. The authors were familiar with both the methodological issues and the topic area. They also had previous experience of working with the tools. There was an explicit procedure for resolving disagreements among authors. All disagreements were resolved by comparing supporting information from each study report, which was divided into two parts of the data collection process (rechecking the document) and a difference in interpretation (resolved via discussion). Unresolved discrepancies were resolved after consulting with a third senior author [28, 29].

## Result

### Literature search

**Fig. 1:**
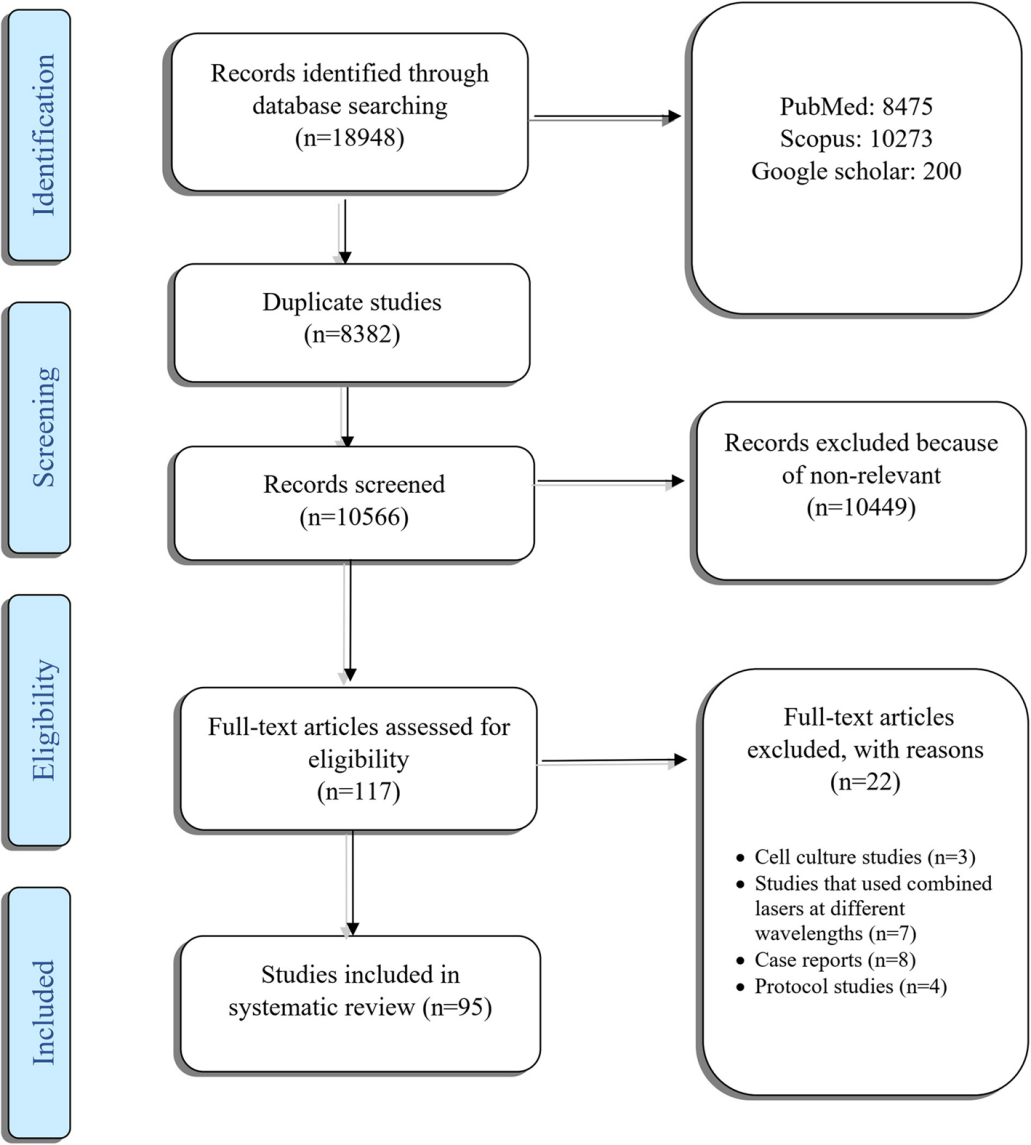
The PRISMA flowchart of the review selection process

The electronic search of the mentioned databases (Google Scholar (*n* = 200), PubMed (8475) and Scopus (10273)), resulted in a total of (18948) studies. After removing duplicated papers (*n* = 8382) and conducting the appraisal process, 117 studies remained for full-text reading. Among these studies, three were excluded because they were conducted in cell culture. Studies that used combined lasers at different wavelengths were also excluded (*n* = 7). Additionally, eight case reports and four protocol studies were excluded. The PRISMA flowchart of the review selection process is illustrated in Fig. 1.

The results yielded 95 studies, which assessed the efficacy of strategies on behavioral and molecular changes in neurological disorders.

The included studies in the first step were divided into two major categories: clinical (human, *n* = 66) and pre-clinical (animal, *n *= 29) studies. Clinical studies were further classified into two main groups including CNS and PNS disorders. The first group was re-classified into neuropsychiatric disorders (*n* = 6), neurodegenerative diseases (*n* = 5), ischemia (*n* = 7), and nerve injury (*n* = 19). The second group contained pain (*n* = 48), paresis (*n* = 3), and neuropathy (*n* = 7). Tables 1 and 2 provide the main characteristics of the included studies outcomes, light source parameters and combination treatments, in central and peripheral nervous system disorders in clinical studies. Since the re-categorizing preclinical studies was not possible due to limitations in the number of articles in each possible section, they were reported in a holistic manner. Table 3 represents the similar data from experimental studies. All included articles addressed the impacts of laser therapy combined with other therapies on neurological disorders.

**Table 1:** Characteristics of the included studies outcomes, light source parameters and combination treatment, in central nervous system disorders in clinical studies

Author	Disease category	Disorder	Sex/Age	Combination therapy	Laser/(LED)	Treatment duration	Key outcomes
Zaizar et al., 2018 [30]	Neuropsychiatric	Fear and anxiety	NM / 18–65	Exposure therapy	1064 nm, 120 J/cm^2^, 0.25 W/cm^2^	NM	Combination therapy improved the outcomes of exposure therapy in pathological fear cases
Zaizar et al., 2023 [31]	Neuropsychiatric	Fear	F / 18 – 65	Exposure therapy	1064 nm, 120 J/cm^2^	NM	Stimulation of dlPFC and vmPFC regions did not enhance exposure therapy outcome
De Marchi et al., 2023 [32]	Neuropsychiatric	Stress	F / 44.81 ± 10.77	Static magnetic field & Pilates	905 nm, 0.085 J/cm^2^ (875 nm, 2.22 J/cm^2^ and 640 nm, 2.77 J/cm^2^)	12 weeks (2/week)	Increased urinary tract`s muscle strength and tone; Improved quality of life and decreased urinary lost
Arakelyan, 2005 [33]	Neurodegenerative	AD	M, F / 73.1	Magnetic field & Light chromo therapies	633 nm	6 courses delivered over 18 months, 15 procedures per course	Combination therapy by magnetic field but not light chromo therapy improved outcomes of ADAS-Cog test in AD patients
Nagy et al., 2021 [34]	Neurodegenerative	AD	M, F / 69.50	Aerobic exercise	650 nm	12 weeks (1/week)	Improved Hb level, MoCa – B basic, quality of life for AD scale and Berg Balance scale scores; Significant reduction in BMI and WHR
Tamae et al., 2020 [35]	Neurodegenerative	PD	NM / 30—80	Vacuum therapy	670, 808 nm	3 weeks (2/week)	Improved muscle pain in parkinsonians; Affected positively the quality of life
Hong et al., 2021 [36]	Neurodegenerative	PD	F / 67.53 ± 8.83	Molecular hydrogen water	940 nm, 6 mW/cm^2^	2 weeks	UPDRS scores began decreasing from the first week, after 1 week of therapy cessation, UPDRS scores slightly increased but the improvement remained significant compared with the baseline
Casalechi et al., 2020 [37]	Ischemia	Stroke	M, F / 45 – 60	Magnetic field	905nm, 0.71 mW/cm^2^ (875 nm, 19.44 mW/cm^2^ and 640 nm, 16.67 mW/cm^2^, 1.27, 3.8, 6.35 J/cm^2^)	4 weeks (4/week)	Positive acute effects on functional mobility in stroke survivors; Improved the 6MWT and TUG tests using a total energy of 30J per site
Ashrafi et al., 2020 [38]	Ischemia	Stroke	M, F / 63.5 ± 14.3	Low frequency electromagnetic field	840 nm	5 days	Combination therapy improved mRS, MMSE and Barthel’s index in stroke cases
Paolillo et al., 2022 [39]	Ischemia	Stroke	M, F / 59 ± 11	Neuromuscular electrical stimulation	660, 808, 980 nm, 360 J/cm^2^	3 months (1/week)	Improved cognitive function, pain relief, greater manual dexterity, physical and social emotional health which lead to better quality of life and well-being
Dumont et al., 2022 [40]	Ischemia	Stroke	M, F / 58.5 ± 10.04	Static magnetic field	905 nm, 0.054, 0.162, 0.271 J/cm^2^ 640 nm, 1.27, 3.8, 6.35 J/cm^2^ 875 nm, 1.48, 4.43, 7.41 J/cm^2^	4 weeks	Improvement was observed in the kinematic variable of the hip in the paretic and non-paretic limbs
da Silva et al., 2020 [41]	Nerve injury	Spinal cord injury	M, F / 36.3 ± 15.1	Physiotherapy	808 nm, 983 J/cm^2^, 4.72 W/cm^2^	4 weeks (3/week)	Leads to better sensory-motor recovery; Increased surface sensitivity, muscle strength, muscle contraction and quality of life

6MWT, the 6-min walk test; *AD* Alzheimer’s disease, *ADAS-Cog* The Alzheimer’s Disease Assessment Scale-cognitive subscale, *BMI* body mass index, *dlPFC* dorsolateral prefrontal cortex, *F* Female, *Hb* Hemoglobin, *M* Male, *MMSE* Mini-Mental State Examination, *MoCa* – B basic, Montreal Cognitive Assessment test for dementia, *mRS* modified Rankin scale, *NM* not mentioned, *PD* Parkinson’s disease, *TUG* Timed Up and Go test, *UPDRS* Unified Parkinson Disease Rating Scale, *vmPFC* ventromedial prefrontal cortex, *WHR* waist–hip ratio

**Table 2:** Characteristics of the included studies outcomes, light source parameters and combination treatment, in peripheral nervous system disorders in clinical studies

Author	Disease category	Disorder	Sex/Age	Combination therapy	Laser/(LED)	Treatment duration	Key outcomes
Aghamohammadi et al., 2012 [42]	Pain	Trigeminal neuralgia	NM / 30–70	Ganglion block	890 nm	6 months 12 sessions	Decreased the severity of pain, dose of carbamazepine; Increased the period of a pain-free state
Ebrahimi et al., 2018 [43]	Pain	Trigeminal neuralgia	M, F / NM	Carbamazepine	810 nm, 6.36 J/cm^2^	3 weeks (3/week)	Decreased pain severity with time
Stergioulas 2007 [44]	Pain	Lateral epicondylitis	M, F / 45.2 ± 2.86	Exercises	904 nm, 2.4 J/cm^2^	8 weeks 12 sessions	A significant decrease of pain at rest, palpation and pain on isometric testing, middle finger test and pain during grip strength test; A significant increase in the wrist range of motion
Celik et al., 2019 [45]	Pain	Lateral epicondylitis	M, F / 48.2 ± 9.4	Exercises	904 nm, 2.4 J/cm^2^	4 weeks (3/week)	Improved elbow extension, shoulder flexion strength, VAS, movement and handgrip strength
Ali et al., 2021 [46]	Pain	lateral epicondylitis	M, F / 44.9 ± 7.3	Ultrasound	808, 915 nm, 5 J/cm^2^	12 sessions	Improved the VAS, DASH score and hand grip-strength
Amanat et al., 2013 [47]	Pain	Orofacial pain	M, F / 47.22	Antidepressants, Anxiolytics, Muscle relaxants, Carbamazepine	980 nm, 12.73 J/cm^2^	3 weeks (3/week)	There was no significant additional level of efficacy for the laser in the management of common orofacial pain based on VAS outcomes
Ceylan et al., 2004 [48]	Pain	Myofascial pain syndrome	M, F / 34.05 ± 8.25	Naproxen sodium, Phenbrobomate	904 nm, 1.44 J/cm^2^	10 days	Increased the VAS values, 5-HIAA and 5-HT + 5-HTP excretion; Reduced pain
Sumen et al., 2015 [49]	Pain	Myofascial pain syndrome	M, F / 41.66 ± 9.26	Exercises	670 nm, 4 J/cm^2^	2 weeks (5/week)	It was found that pain (according VAS Index) was significantly lower in combination therapy group in comparison to exercise only
El-sharkawy et al., 2018 [50]	Pain	Myofascial pain syndrome	M, F / NM	Ultrasound, Hot pack, Exercise	905, 808 nm, 16 J/cm^2^	4 weeks (3/week)	Increased the quality of life, pressure pain threshold for temporomandibular join, masseter and anterior temporalis muscles
Mansourian et al., 2019 [51]	Pain	Myofascial pain syndrome	M, F / 18–60	Fluoxetine, Clonazepam	810 nm, 2 J/cm^2^	5 weeks (2/week)	Improved pain and limitation in lateral movements
Gur et al., 2003 [52]	Pain	Chronic low back pain	M, F / 35.2 ± 10.51	Exercise	1 J/cm^2^	4 weeks (5/week)	Laser therapy seemed to be an effective method in reducing pain and functional disability. However, does not bring any additional benefits to exercise therapy
Djavid et al., 2007 [53]	Pain	Chronic low back pain	M, F / 38	Exercise	810 nm, 27 J/cm^2^	6 weeks (2/week)	No greater effect of laser therapy plus exercise compared with exercise for any outcome; Reduced pain; Increased lumbar range of movement on the Schober Test and active flexion; Reduced disability
Ammar 2015 [54]	Pain	Chronic low back pain	M, F / 42.1 ± 12.8	Exercise	850 nm	6 weeks (2/week)	Improved functional disability, pain and lumbar ROM
Koldaş Doğan et al., 2017 [55]	Pain	Chronic low back pain	M, F / 52.14 ± 12.13	Hot pack	850 nm, 10 J/cm^2^ 650, 785, 980 nm, 3 J/cm^2^	3 weeks (5/week)	Improved pain severity, patient’s and physician’s global assessment, ROM and MODQ scores; Laser therapy provided more improvements in lateral flexion measurements and disability of the patients
Mohammad Ezz El Dien et al., 2007 [56]	Pain	Primary periarthritis shoulder	M, F / 49.2 ± 5.9	Electromagnetic field, Exercise	880 nm, 1 J/cm^2^	2 months (3/week)	Improved all shoulder parameters (pain, tenderness, range of motion and function)
Otadi et al., 2012 [57]	Pain	Shoulder tendonitis	F / 49.48 ± 8.5	Ultrasound, Exercise	830 nm, 1 J/cm^2^	10 sessions (3/week)	Improved VAS, TSS, CMS and the muscle strengths
Eslamian et al., 2012 [58]	Pain	Rotator cuff tendinitis	M, F / 50.16 ± 12.10	Physiotherapy	830 nm, 4 J/cm^2^	10 sessions (3/week)	Improved pain (reduction in VAS average) and shoulder disability problems; Improved the patient’s function; No additional advantages were detected in increasing shoulder joint range of motion in comparison to other physical agents
Dogan et al., 2010 [59]	Pain	Subacromial impingement syndrome	M, F / 53.59 ± 11.34	Cold pack	850 nm, 5 J/cm^2^	14 sessions (5/week)	Improved pain severity, range of motion except internal and external rotation and SPADI scores
Abrisham et al., 2011 [60]	Pain	Subacromial syndrome	M, F / 52.2 ± 5.7	Exercise	890 nm, 2–4 J/cm^2^	2 weeks (5/week)	Significant post-treatment improvements were achieved in all parameters, in all movements; There was a substantial difference between the groups in VAS scores; Improved the shoulder ROM
Pekyavas et al., 2016 [61]	Pain	Subacromial impingement syndrome	NM / 51.1 ± 14.3	Manual therapy, Kinesio taping, Exercise	1064 nm	15 sessions (3/week)	Minimized pain and disability; Increased ROM and SPADI
Alfredo et al., 2021 [62]	Pain	Subacromial impingement syndrome	NM / 51.9 ± 8.7	Exercise	904 nm	8 weeks (3/week)	Improved shoulder function; Reduced pain intensity and medication intake
Ökmen et al., 2017 [63]	Pain	Chronic shoulder pain	M, F / 53	Exercise	1064 nm, 100 J/cm^2^	2 weeks (7/week)	Compared to the values of PRT and PST at months 1, 3, and 6, VAS, SPADI, and NHP values were lower
Teixeira et al., 2022 [64]	Pain	Chronic neck/shoulder pain	M, F / 32.78 ± 9.99	Magnetic field	905, 875, 640 nm	3 weeks (2/week)	Reduced pain intensity (reduction in VAS) in all time points tested; There was no difference in the ROM outcomes
Kolu et al., 2018 [65]	Pain	Chronic lumbar radiculopathy	M, F / 53.40 ± 10.57	Hot pack, Exercise	12, 120 J/cm^2^	2 weeks (5/week)	Decreased pain variation and functionality (VAS and ODI)
Stasinopoulos et al., 2009 [66]	Pain	Lateral elbow tendinopathy	NM / 18 ≤	Exercise	904 nm, 130 mW/cm^2^	4 weeks (3/week)	Decline in pain; Increase in function compared with baseline has been observed
Liu et al., 2014 [67]	Pain	Patellar tendinopathy	M / 18–23	Exercise	810 nm, 1592 mW/cm^2^	4 weeks (6/week)	Reduced pain (VAS); Improved function capacity of knee, muscle strength and endurance
Stergioulas et al., 2008 [68]	Pain	Chronic achilles tendinopathy	M, F / 30.1 ± 4.8	Exercise	820 nm, 60 mW/cm^2^	8 weeks 12 sessions	Combination therapy accelerates clinical recovery as tested by VAS; Power densities below 100 mW/cm^2^ seems to be important for obtaining good results
Saayman et al., 2011 [69]	Pain	Cervical facet dysfunction	F / 18–40	Chiropractic joint manipulation therapy	830 nm, 151 mW/cm^2^	3 weeks (2/week)	The combination therapy was more effective than either of the 2 on their own; Pain disability in everyday life, lateral flexion, and rotation was the main outcomes
Gu et al., 2017 [70]	Pain	Cervical spondylosis	M, F / 35—71	Ozone therapy	NM	NM	Decreased preoperative neck and shoulder pain (VAS score) at 1 month period
Venosa et al., 2019 [71]	Pain	Cervical spondylosis	M, F / 49.76	Exercise	1064 nm	6 weeks (2/week)	Increased cervical ROM; Reduced pain; There was a significant difference in NDI scores; Analgesic effects; Improved function in patients affected by cervical spondylosis
Yilmaz et al., 2020 [72]	Pain	Cervical pain	M, F / 18–60	Exercise	1064 nm, 5 J/cm^2^	4 weeks (5/week)	Improved cervical range of motion and quality of life by reducing pain (ROM, VAS and NPADS values)
De Carli et al., 2013 [73]	Pain	Temporomandibular joint pain	NM	Piroxicam	808 nm, 100 J/cm^2^	10 days	Combination therapy was not more effective than single therapies (evaluated by VAS)
Elgohary et al., 2018 [74]	Pain	Temporomandibular joint pain	M, F / 60.75 ± 5.09	Exercise	950 nm, 7.6 J/cm^2^	4 weeks (5/week)	Improvement in VAS, VCS and UW-QOL questionnaire results
Brochado et al., 2018 [75]	Pain	Temporomandibular joint pain	M, F / 46.5 ± 14.4	Manual therapy	808 nm, 13.3 J/cm^2^	4 weeks (3/week)	Reduced depression symptoms, anxiety symptoms and physical symptoms; Promoted pain relief; Improved mandibular function and jaw disabilities
Ahmad et al., 2018 [76]	Pain	Temporomandibular joint pain	M, F / 37.56 ± 8.26	Ultrasound, Hot pack, Exercise	905, 808 nm, 16 J/cm^2^	4 weeks (3/week)	Decreased limitations in daily functions; Increased pressure pain threshold for masseter and anterior temporalis muscles
Panhoca et al., 2019 [77]	Pain	Temporomandibular joint pain	M, F / 23—66	Ultrasound	808 nm, 32.832 J/cm^2^	4 weeks (2/week)	Synergistic treatment was effective in improving the oral health-related quality of life (assessed by the Oral Health Impact Profile)
Panhóca et al., 2021 [78]	Pain	Temporomandibular joint pain	M, F / 18—55	Ultrasound	808 nm, 684 J/cm^2^	4 weeks (2/week)	Laser combined with ultrasound are effective in the treatment of pain as assessed by analogue pain scale; Assessment of range of motion and assessment of quality of life
Panhóca et al., 2021 [78]	Pain	Temporomandibular joint pain	M, F / 18—55	Vacuum therapy	808 nm, 684 J/cm^2^	4 weeks (2/week)	Laser combined with vacuum are effective in the treatment of pain as assessed by analogue pain scale; Assessment of range of motion and assessment of quality of life
Dias et al., 2022 [79]	Pain	Temporomandibular joint pain	M, F / 32.16 ± 8.60	Orofacial myofunctional therapy	830 nm, 51 and 34 J/cm^2^	13 sessions	Improved the degree of pain (VAS) and self-perception of the OHQOL
Matsutani et al., 2007 [80]	Pain	Fibromyalgia	F / 44	Exercise	830 nm 3 J/cm^2^	5 weeks (2/week)	Pain reduction; Higher pain threshold at tender points; Lower mean FIQ scores; Higher SF-36 mean scores
da Silva et al., 2018 [81]	Pain	Fibromyalgia	F / ≥ 35	Exercise	905 nm, 0.75 J/cm^2^ (640 nm, 5 J/cm^2^ and 875 nm, 5.83 J/cm^2^)	10 weeks (2/week)	Improved pain threshold in several tender points; A more substantial effect was noticed for the combined therapy; Pain relief was accomplished by improving VAS and FIQ scores as well as quality of life
Germano Maciel et al., 2018 [82]	Pain	Fibromyalgia	F / 30—50	Exercise	808 nm, 142.85 J/cm^2^	8 weeks (3/week)	Reduced pain; Improved function, muscular performance, depression, and quality of life; The benefic effects of functional exercise were not improved by combination with LLLT
Aquino Junior et al., 2021 [83]	Pain	Fibromyalgia	F / 30—65	Ultrasound	660 nm	2 to 10 weekly sessions	Combination therapy was more efficient in improvement in the pain of fibromyalgia as tested by FIQ and VAS
Paolillo et al., 2015 [84]	Pain	Osteoarthritis	F / 68 ± 6	Ultrasound, Exercise	808 nm, 7 J/cm^2^	3 months (1/week)	Grip strength did not differ; Significant decrease of the pain sensitivity
Gavish et al., 2021 [85]	Pain	Knee pain	M, F / > 18	Physiotherapy	810 nm, 142.5 and 180 J/cm^2^ (660/850 nm, 3 J/cm^2^)	4 weeks (2/week)	Reduced pain (VAS); Improved the Kujala score
Murakami et al., 1993 [86]	Paresis	Facial palsy	M, F / 41.8 ± 4.7	Ganglion block	830 nm	NM	The combination therapy showed a similar overall recovery of facial palsy to ganglion block
Yamada et al., 1995 [87]	Paresis	Facial palsy	NM / 45.1 ± 14.0	Corticosteroid	830 nm 36.7, 38.2 and 127.4 J/cm^2^	3–10 weeks	Combination therapy is an ideal adjunct treatment in cases that corticosteroid therapy is mineable
Ordahan 2017 [88]	Paresis	Bell’s palsy	M, F / 41 ± 9.7	Exercise	830 nm, 10 J/cm^2^	6 weeks (3/week)	Improved functional facial movements through the FDI; Decreased recovery times for patients
Naeser et al., 2002 [89]	Neuropathy	Carpal tunnel syndrome	M, F / 53.5	Transcutaneous electric nerve stimulation	632.8, 904 nm, 1.81 J/cm^2^	3 to 4 weeks (3/week)	Significant decreases in MPQ score, median nerve Sensory latency, and Phalen and Tinel signs
Dincer et al., 2009 [90]	Neuropathy	Carpal tunnel syndrome	F / 52.2 ± 9.1	Splinting	904 nm, 1 J/cm^2^	2 weeks (5/week)	Reduced symptom severity and pain; Increased patient satisfaction using BQ SSS, BQ FSS, VAS, ENMG testing
Yagci et al., 2009 [91]	Neuropathy	Carpal tunnel syndrome	F / 49.47 ± 6.32	Splinting	830 nm	10 sessions	Improved both clinical and NCS parameters (median motor nerve distal latency, median sensory nerve conduction velocities, BQ SSS, and BQ FCS); Provided better outcomes on NCS
Fusakul et al., 2014 [92]	Neuropathy	Carpal tunnel syndrome	M, F / 50.70 ± 1.39	Splinting	810 nm	5 weeks (3/week)	Improved hand grip strength, distal motor latency of the median nerve and electroneurophysiological parameters at 5 and 12-week follow-up
Tabatabai et al., 2016 [93]	Neuropathy	Carpal tunnel syndrome	M, F / 48.60	Transcutaneous electrical nerve stimulation	808 nm, 6.5 J/cm^2^	2 weeks (5/week)	Reduced the mean scores of MPQ, VAS, pain severity, and DASH questionnaires
Güner et al., 2018 [94]	Neuropathy	Carpal tunnel syndrome	F / 44.33 ± 9.21	Kinesiotaping	685 nm, 5 J/cm^2^	3 weeks (5/week)	Improved VNS daytime, VNS night, FPS, HGS, BQ SSS, BQ FCS parameters at 3th and 12th weeks compared to before treatment; Improved mMA, mSNCV, and mSDL parameters at the 12th week (from ENMG parameters)
Bartkowiak et al., 2019 [95]	Neuropathy	Carpal tunnel syndrome	M, F / 46.8 ± 10.8	Exercise	830 nm, 9 J/cm^2^	2 weeks (5/week)	Declined sensory impairments and pain; Improved hand grip strength, VAS, Boston Questionnaire results, CTS SSS and CTS FSS

*5-HIAA* 5-hydroxy indole acetic acid *5-HT* serotonin, *5-HTTP* 5-hydroxy tryptophan, *BQ FCS* Boston Questionnaire functional capacity scale, *BQ FSS* Boston Questionnaire functional status scale, *BQ SSS* Boston Questionnaire symptom severity scale, *CMS* Constant Murley Score, *CTS FSS* The carpal tunnel syndrome functional status scale, *CTS SSS* The carpal tunnel syndrome symptom severity scale, *DASH* Disabilities of the Arm, Shoulder and Hand, *ENMG* Electroneuromyography, *F* Female, *FDI* facial disability index, *FIQ* Fibromyalgia Impact Questionnaire, *FPS* Finger pinch strength, *HGS* Hand grip strength, *LLLT* Low level laser therapy, *M* Male, *mMA* motor amplitude, *MODQ* Modified Oswestry Disability Questionnaire, *MPQ* McGill Pain Questionnaire, *mSDL* the sensory distal latency, *mSNCV* the sensory conduction velocity, *NCS* nerve conduction study, *NDI* Neck disability index, *NHP* Nottingham Health Profile, *NM* not mentioned, *NPADS* Neck pain and disability scale, *ODI* Oswestry Disability Index, *OHQOL* Oral health quality of life, *PRT* pretreatment, *PST* posttreatment, *ROM* range of motion, *SF-36* 36-item Short-Form Health Survey, *SPADI* Shoulder Pain and Disability Index, *TSS* Tenderness Severity Scale, *UW-QOL* University of Washington Quality of Life questionnaire, *VAS* visual analogue scale, *VCS* Vernier caliper scale, *VNS* visual numeric pain scale

**Table 3:** Characteristics of the included studies outcomes, light source parameters and combination treatment, in experimental studies

Author	Disease category	Disorder	Species	Sex/Age	Combination therapy	Laser	Treatment duration	Key outcomes
Salehpour et al., 2019 [96]	Neuropsychiatric	Depression	BALB/c mice	M / Adult	CoQ_10_	810 nm, 33.3 J/cm^2^, 6.66 W/cm^2^	5 days	Antidepressant-like effects; Decreased lipid peroxidation, corticosterone, TNF-α, and IL-6; Enhanced total TAC, GSH levels, GPx and SOD activities in HIP and PFC; The inflammatory response in the HIP and PFC was suppressed, as indicated by decreased NF-kB, p38, and JNK levels; Down-regulated intrinsic apoptosis biomarkers, BAX, Bcl-2, cytochrome c release, and caspase-3 and -9
Meynaghizadeh-Zargar et al., 2020 [97]	Neuropsychiatric	Chronic mild stress	BALB/c mice	M / 8 weeks	Methylene blue	810 nm, 8 J/cm^2^, 4.75 W/cm^2^	4 weeks (3/week)	Anxiolytic effects; Therapeutic effects on mitochondrial dysfunction, learning and memory impairments; Decreased serum cortisol levels, NO production, ROS production, SOD; Increased TAC, GPx
Farazi et al., 2022 [98]	Neuropsychiatric	Depression	BALB/c mice	M / Adult	Environmental enrichment	810 nm, 8 J/cm^2^, 4.75 W/cm^2^	14 days	Antidepressant-like effects; Up-regulated hippocampal BDNF/TrkB/CREB signaling pathway
Moges et al., 2009 [99]	Neurodegenerative	Amyotrophic lateral sclerosis	G93A SOD1 Transgenic mice	NM / 51 days	Riboflavin	810 nm, 12 J/cm^2^	(3/week)	The lack of significant improvement in survival and motor performance indicates interventions were ineffective in altering disease progression
Lapchak et al., 2008 [100]	Ischemia	Embolic stroke model	New Zealand white rabbits	M / NM	Tissue plasminogen activator	808 nm, 10 mW/cm^2^	NM	Near-infrared laser therapy may be administered safely either alone or in combination with tPA because neither treatment affected hemorrhage incidence or volume
Li et al., 2014 [101]	Ischemia	Hypoxic-ischemic brain damage	Sprague–Dawley rats	M, F / 3 months	Mesenchymal stem cell	660 nm, 60 mW/cm^2^	7 days	Diode irradiation promotes migration of the transplanted bone marrow mesenchymal stem cells
Salehpour et al., 2019 [102]	Ischemia	Ischemia	BALB/c mice	M / Adult	CoQ_10_	810 nm, 33.3 J/cm^2^, 6.66 W/cm^2^	14 days	Improved spatial and episodic memory; Lowered ROS levels; Increased ATP and general mitochondrial activity as well as biomarkers of mitochondrial biogenesis including SIRT1, PGC-1α, NRF1, and TFAM; Decreased inflammatory responsiveness, iNOS, TNF-α and IL-1β levels
Menovsky et al., 2003 [103]	Nerve injury	Sciatic nerve crush	Wistar rats	M	Solder and suture materials	CO2 laser	NM	Leads to optimal early histological results and least foreign-body reaction at the repair site
Duke et al., 2012 [104]	Nerve injury	Sciatic nerve crush	Sprague–Dawley rats	M	Electrical stimulation	1875 nm	NM	Reduces the laser power requirements and mitigates the risk of thermal damage while maintaining spatial selectivity
Dias et al., 2013 [105]	Nerve injury	Sciatic nerve crush	Wistar rats	M	Natural latex protein	780 nm, 15 J/cm^2^, 0.75 W/cm^2^	6 sessions (Alternate days)	Improved the myelin density and morphological characteristics; The capillary density and ultrastructural characteristics were similar to the control group
Yang et al., 2016 [106]	Nerve injury	Sciatic nerve crush	Sprague–Dawley rats	M / Adult	Mesenchymal stem cells	660 nm, 9 J/cm^2^	7 days	Provided greater functional recovery; Potentiated recovery in SFI, VA and AA; Increased electrophysiological function and expression of S100 immunoreactivity; Reduced the inflammatory cells
Dias et al., 2015 [107]	Nerve injury	Sciatic nerve crush	Wistar rats	M	Latex protein	780 nm, 15 J/cm^2^, 0.75 W/cm^2^	6 sessions (Alternate days)	Improvement of the nerve characteristics including the morphometric and ultrastructural characteristics of nerve fibers
Muniz et al., 2015 [108]	Nerve injury	Sciatic nerve crush	Wistar rats	M	Natural latex protein	780 nm, 15 J/cm^2^, 0.75 W/cm^2^	12 days (6/48 h)	Improved muscle fiber atrophy; Increased light fiber area and reduced dark fiber area
de Souza et al., 2018 [109]	Nerve injury	Sciatic nerve crush	Swiss mice	M / Adult	Dexamethasone	660 nm, 10 J/cm^2^	28 days	Improved nerve regeneration through the SSI and SFI assessments
Dias et al., 2019 [110]	Nerve injury	Sciatic nerve crush	Wistar rats	M / 2 months	Natural latex protein	780 nm, 15 J/cm^2^, 0.75 W/cm^2^	6 sessions (Alternate days)	Improved nerve fiber regeneration; Reduced the number, density, diameter and organization of nerve fibers; Increased NGF, VEGF
de Souza et al., 2021 [111]	Nerve injury	Sciatic nerve crush	Swiss mice	M / 3 months	Simvastatin	660 nm, 10 J/cm^2^	28 days	Sciatic functional index, thermal heat hyperalgesia, mechanical hyperalgesia, and thermographic were evaluated; The results showed that PBM alone was more effective compared to Simvastatin alone or combination
Souza et al., 2013 [112]	Nerve injury	Spinal cord injury	Wistar rats	M / 20—21 weeks	Monosialoganglioside	NM	42 days	Combination therapy shows no superior functional, neurological or histological results
Janzadeh et al., 2017 [113]	Nerve injury	Spinal cord injury	Wistar rats	M / Adult	Chondroitinase ABC	660 nm, 0.5 J/cm^2^, 0.819 W/cm^2^	14 days	Improved motor function recovery, myelination and number of axons; Decreased GSK3β, CSPG, and AQP4 expression
Pedram et al., 2018 [114]	Nerve injury	Spinal cord injury	Fischer-344, Wistar rats	M / 8 – 12 weeks	Meloxicam	810 nm, 6 J/cm^2^, 200 mW/cm^2^	2 weeks	Increased BBB test results; Histological findings revealed no significant difference between all study groups
Sarveazad et al., 2019 [115]	Nerve injury	Spinal cord injury	Wistar rats	M / Adult	Human adipose derived stem cells	660 nm	2 weeks	Improved the motor function, SCI-induced allodynia and hyperalgesia; Increased the GDNF, GABA receptors and Gad65 expression level; Reduced the expression of GSK3β, IL-6, AQP4
Janzadeh et al., 2020 [116]	Nerve injury	Spinal cord injury	Wistar rats	M / Adult	Chondroitinase ABC	660 nm, 22.8 J/cm^2^, 500 mW/cm^2^	2 weeks	Reduced allodynia and thermal hyperalgesia; Improved functional recovery; Did not reduce mechanical hyperalgesia; Decreased BDNF and IL-6; Increased Gad65 and GDNF; Reduced neuropathic pain; Improved movement
Chen et al., 2021 [117]	Nerve injury	Spinal cord injury	Sprague–Dawley rats	M / 12 weeks	Human umbilical cord mesenchymal stem cells	630 nm, 100 mW/cm^2^	14 days	Improved neurofilament structure and arrangement; Promoted motor function and neuronal recovery; Increased the expression of NF‐200, glial fibrillary acidic protein in the damaged area and the BBB scores; Nissl bodies were more numerous and the nerve fibers were longer and thicker; Reduced lesions volume and secondary damage; Promoted functional recovery
Dong et al., 2015 [118]	Nerve injury	Traumatic brain injury	C57BL/6 mice	NM / 8 weeks	Lactate / Pyruvate	810 nm, 36 J/cm^2^, 150 mW/cm^2^	NM	Retained memory and learning activities of injured mice to a normal level; Low levels of glycolysis; Increased ATP; Reduced formation of ROS and apoptosis in neurons
Buchaim et al., 2016 [119]	Nerve injury	Facial nerve injury	Wistar rats	M / 60 days	Heterologous fibrin sealant	830 nm, 6 J/cm^2^, 258.6 mW/cm^2^	5 weeks (3/week)	Combination group presented the closest results to the control, in all nerve morphometry indexes (regeneration), except in the axon area
de Oliveira Rosso et al., 2017 [120]	Nerve injury	Facial nerve injury	Wistar rats	M / 80 days	Heterologous fibrin sealant	830 nm, 6.2 J/cm^2^, 0.26 W/cm^2^	5 weeks (3/week)	A significant difference in the fiber nerve area; The functional recovery of whisker movement; Accelerated morphological and functional nerve repair
Jameie et al., 2014 [121]	Pain	Neuropathic pain (Chronic constriction injury model)	Wistar rat	M / Adult	CoQ_10_	980 nm, 4 J/cm^2^, 0.248 W/cm^2^	2 weeks	Cellular and molecular synergism on pain relief; Increased thermal and mechanical sense thresholds
Noma et al., 2020 [122]	Pain	Neuropathic pain (Trigeminal nerve injury)	Sprague–Dawley Wistar rat	M / 5–6 weeks	Oxytocin	810 nm, 0.1 W	3 days	The expanded area of cortical excitation caused by model was suppressed by combination therapy but not by each treatment alone; Combined application is effective in relieving the neuropathic pain
Martins et al., 2020 [123]	Pain	Orofacial pain	Wistar rats	M / 2 months	Vitamins B complex	904 nm, 6 J/cm^2^	10 sessions	Maximal antiallodynic effect; Improved the nociceptive behavior; Down-regulated expression of GFAP, Iba-1, IL-1β, IL-6 and TNF-α; Increased IL-10 expression
de Freitas Dutra Júnior et al., 2022 [124]	Pain	Calcaneus tendon injury	Wistar rats	NM / 60 days	Heterologous fibrin biopolymer	660 nm, 6 J/cm^2^, 1 W/cm^2^	3 weeks (1/week)	Reduced the volume of the edema; Stimulate the repair process; Tenocyte proliferation, granulation tissue and collagen formation were observed in the PTCT area

*AA* ankle angle, *AQP4* aquaporin 4, *ATP* adenosine triphosphate, *BAX* Bcl-2 associated X protein, *BBB* Basso-Beattie-Bresnahan test, *Bcl-2* B-cell lymphoma 2, *BDNF* brain-derived neurotrophic factor, *CoQ*_*10*_ Coenzyme Q_10_, *CREB* cAMP response element-binding, *CSPG* chondroitin sulfate proteoglycan, *F* Female, *GABA* Gamma-aminobutyric acid, *Gad65* Glutamic acid decarboxylase65, *GDNF* glial-derived neurotrophic factor, *GFAP* glial fibrillary acid protein, *GPx* glutathione peroxidase, *GSH* glutathione, *GSK3β* glycogen synthase kinase-3β, *HIP* hippocampus, *Iba-1* ionized calcium binding adaptor molecule 1, *IL-10* Interleukin-10, *IL-1β* Interleukin-1β, *IL-6* interleukin-6, *iNOS* inducible NO synthase, *JNK* c-Jun aminoterminal kinases, *M* Male, *NF‐200* neurofilament 200, *NF-kB* nuclear factor-Kb, *NGF* nerve growth factor, *NM* not mentioned, *NO* nitric oxide, *NRF1* nuclear respiratory factor 1, *PFC* prefrontal cortex, *PGC1-α* peroxisome proliferator-activated receptor gamma coactivator-1 alpha, *PTCT* partial transection of the calcaneus tendon, *ROS* reactive oxygen species, *SFI* Sciatic Functional Index, *SIRT1* silent mating-type information regulation 2 homolog 1, *SOD* superoxide dismutase, *SSI* Sciatic Static Index, *TAC* total antioxidant capacity, *TFAM* mitochondrial transcription factor A, *TNF-α* tumor necrosis factor-alpha, *tPA* tissue plasminogen activator, *TrkB* tyrosine receptor kinase B, *VA* vertical activity of locomotion, *VEGF* vascular endothelium growth factor

### Study characteristics

The wavelength, power/energy density (irradiance and fluence), mode of application (pulsed wave or continuous wave), and treatment frequency were the most important factors affecting the outcomes. Red to far-infrared lasers at a range of wavelengths from 630 to 1875 nm were widely used, as opposed to LEDs and CO2 lasers. The included protocols had an energy density of up to 983 J/cm^2^. According to the findings of this study, laser therapy was combined with other treatment approaches such as pharmacotherapy, exercise, environmental enrichment, exposure therapy, physiotherapy, ultrasound, mesenchymal stem cells, etc. The duration of treatment varied from 3 days to 18 months. Almost all studies showed positive effects of PBM-combined therapies on various neurological disorders.

### Study quality and risk of bias

**Fig. 2:**
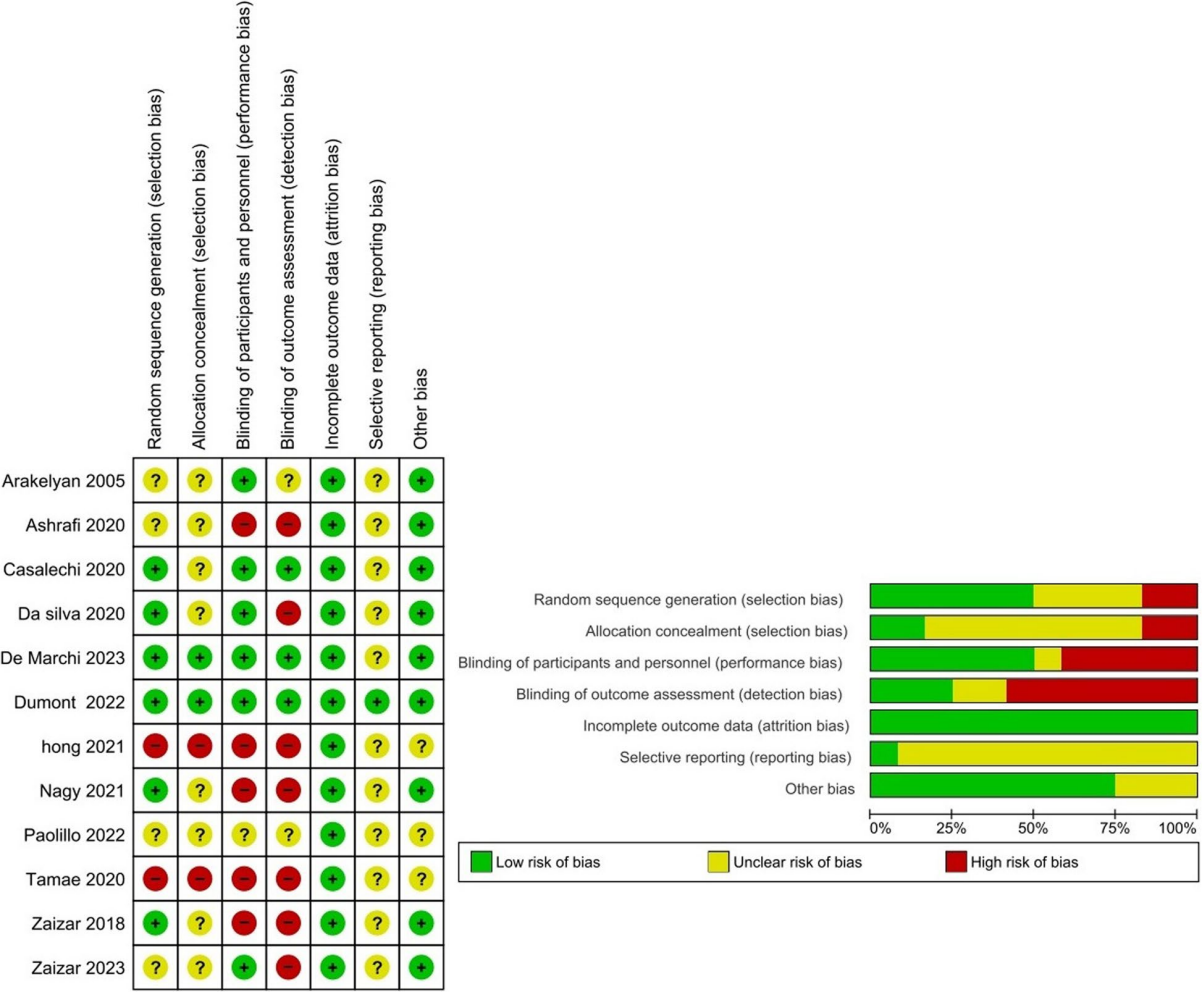
Risk of bias (RoB) assessment using Cochrane RoB tool (included CNS studies). Left panel shows RoB summary showing each RoB item for each included study. Right panel shows RoB graph showing each RoB item presented as percentages across all included studies. In this color-coded ranking, the green color represents a low RoB and red high RoB

**Fig. 3:**
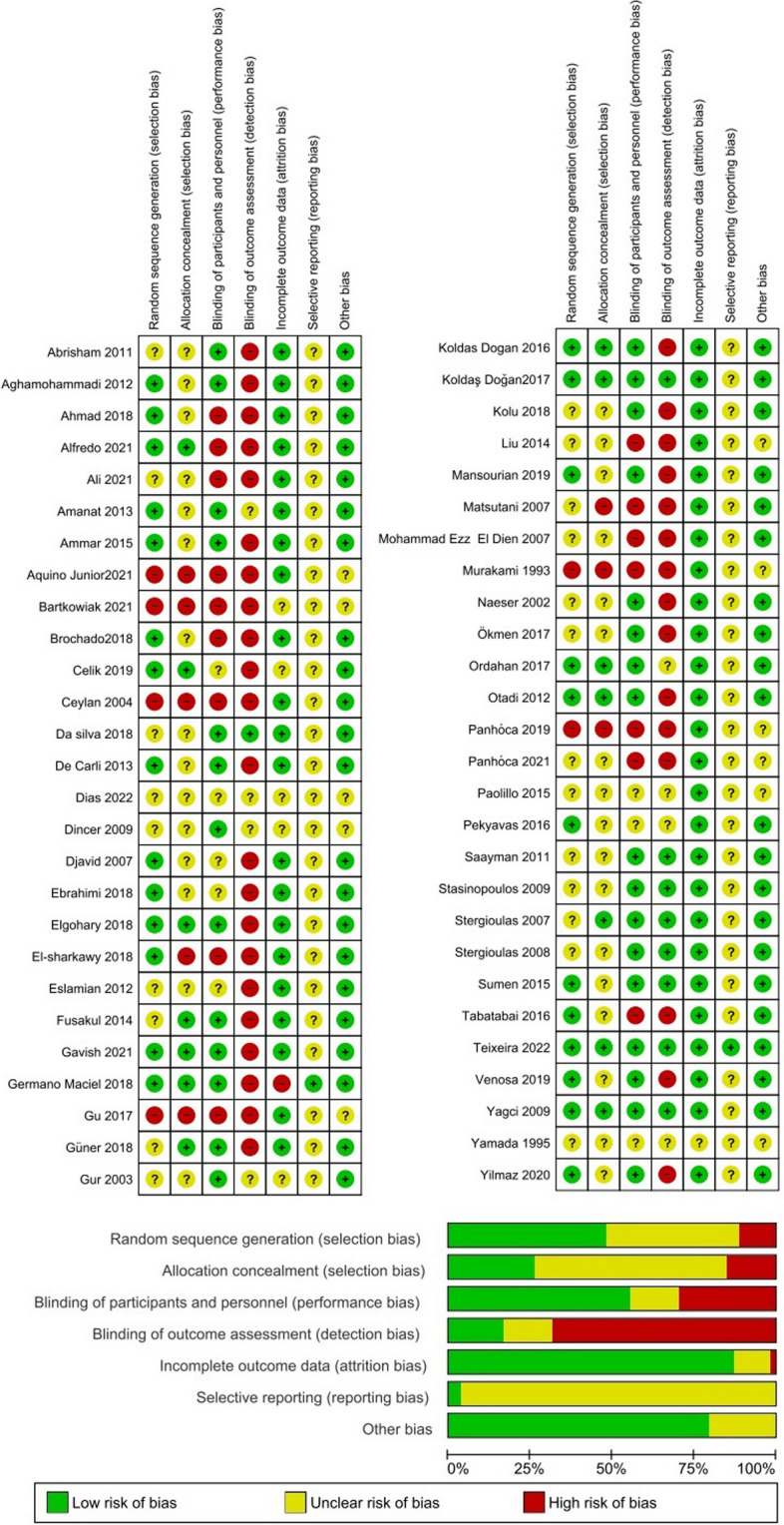
Risk of bias (RoB) assessment using Cochrane RoB tool (included PNS studies). Top panel shows RoB summary showing each RoB item for each included study. Bottom panel shows RoB graph showing each RoB item presented as percentages across all included studies. In this color-coded ranking, the green color represents a low RoB and red high RoB

The Cochrane Collaboration’s tool showed that the majority of CNS studies were not blinded, and the allocation concealment rate was low in these studies. Accordingly, selection, and detection bias were apparent in these studies. The details of the quality assessment are presented in Figs. 2 and 3. The CAMARADES checklist was utilized in the quality assessment of animal studies which showed that almost all studies were qualified (Table 4). All of the articles had been published in peer-reviewed journals and reported details of the animal model, anesthetic use, compliance with animal welfare, and a statement of potential conflicts of interest. Random allocation to groups was reported in 18 (62%) studies. Nine (31%) studies reported blinded induction of the model. Only one study reported a sample size calculation and 10 (34%) studies reported blinded assessment of the outcome.

**Table 4:** The methodological quality of individual animal studies using the CAMARADES checklist

Authors	Q1	Q2	Q3	Q4	Q5	Q6	Q7	Q8	Q9	Q10
		**20**	**18**	**9**	**10**			**1**		
**de Oliveira Rosso, 2017** [120]	Yes	Yes	Yes	Yes	Yes	Yes	Yes	No	Yes	Yes
**Menovsky, 2003** [103]	Yes	Yes	Yes	No	No	Yes	Yes	No	Yes	Yes
**Buchaim, 2016** [119]	Yes	Yes	Yes	No	No	Yes	Yes	No	Yes	Yes
**Chen, 2021** [117]	Yes	Yes	Yes	Yes	Yes	Yes	Yes	No	Yes	Yes
**de Freitas Dutra Júnior, 2022** [124]	Yes	Yes	Yes	Yes	Yes	Yes	Yes	Yes	Yes	Yes
**de Souza, 2021** [111]	Yes	Yes	Yes	No	No	Yes	Yes	No	Yes	Yes
**de Souza, 2018** [109]	Yes	No	No	No	No	Yes	Yes	No	Yes	Yes
**Dias, 2013** [105]	Yes	Yes	No	No	No	Yes	Yes	No	Yes	Yes
**Dias, 2015** [107]	Yes	Yes	No	No	No	Yes	Yes	No	Yes	Yes
**Dias, 2019** [110]	Yes	Yes	No	No	No	Yes	Yes	No	Yes	Yes
**Dong, 2015** [118]	Yes	Yes	No	No	No	Yes	Yes	No	Yes	Yes
**Duke, 2012** [104]	Yes	No	No	No	No	Yes	Yes	No	Yes	Yes
**Farazi, 2022** [98]	Yes	Yes	Yes	No	No	Yes	Yes	No	Yes	Yes
**Jameie, 2014** [121]	Yes	No	No	No	No	Yes	Yes	No	Yes	Yes
**Janzadeh, 2020** [116]	Yes	No	Yes	Yes	Yes	Yes	Yes	No	Yes	Yes
**Janzadeh, 2017** [113]	Yes	No	Yes	Yes	Yes	Yes	Yes	No	Yes	Yes
**Lapchak, 2008** [100]	Yes	Yes	Yes	No	Yes	Yes	Yes	No	Yes	Yes
**Martins, 2020** [123]	Yes	No	No	No	No	Yes	Yes	No	Yes	Yes
**Meynaghizadeh-Zargar, 2020** [97]	Yes	Yes	Yes	No	No	Yes	Yes	No	Yes	Yes
**Moges, 2009** [99]	Yes	No	No	No	No	Yes	Yes	No	Yes	Yes
**Muniz, 2015** [108]	Yes	Yes	Yes	No	No	Yes	Yes	No	Yes	Yes
**Noma, 2020** [122]	Yes	Yes	Yes	No	No	Yes	Yes	No	Yes	Yes
**Pedram, 2018** [114]	Yes	Yes	Yes	Yes	Yes	Yes	Yes	No	Yes	Yes
**Salehpour, 2019** [96]	Yes	Yes	Yes	Yes	Yes	Yes	Yes	No	Yes	Yes
**Salehpour, 2019** [102]	Yes	Yes	Yes	No	No	Yes	Yes	No	Yes	Yes
**Sarveazad, 2019** [115]	Yes	Yes	Yes	No	No	Yes	Yes	No	Yes	Yes
**Souza, 2013** [112]	Yes	No	No	Yes	Yes	Yes	Yes	No	Yes	Yes
**Li, 2014** [101]	Yes	No	No	No	No	Yes	Yes	No	Yes	Yes
**Yang, 2016** [106]	Yes	Yes	Yes	Yes	Yes	Yes	Yes	No	Yes	Yes

Studies fulfilling the criteria of (Q1) peer-reviewed publication; (Q2) control of temperature; (Q3) random allocation; (Q4) blinded induction; (Q5) blinded assessment of outcome; (Q6) use of anesthetic; (Q7) animal model; (Q8) sample size calculation; (Q9) compliance with animal welfare regulations; and (Q10) statement of potential conflict of interests

## Discussion

This systematic review sought to assess whether the integration of photobiomodulation (PBM) with other treatment strategies yielded additional advantages in the management of neurological and neuropsychological disorders, as compared to administering these treatments separately.

### Central Nervous System (CNS)

#### Neuropsychiatric disorders

Zaizar et al. [30, 31] showed that the concurrent use of transcranial infrared laser and exposure therapy reduced fear in individuals with pathological fear. The study findings demonstrated that the combination of PBM with a static magnetic field and Pilates, a therapeutic approach for stress incontinence, resulted in enhanced muscle strength and reduced urinary loss [32]. In addition, certain studies have found that the concurrent use of transcranial PBM with pharmaceutical interventions, such as coenzyme Q10 and methylene blue, can reduce anxiety by counteracting oxidative stress, neuroinflammation, and neuronal apoptosis [96, 97]. Furthermore, the concurrent use of transcranial PBM and a stimulating environment has demonstrated a substantial elevation in hippocampal levels of BDNF, TrkB levels, and the p-CREB/CREB ratio, alongside a reduction in depressive behaviors [98].

#### Neurodegenerative diseases

Arakelyan et al. showed that the combination of low-level laser therapy (LLLT), magnetic field therapy, and light chromotherapy was more effective than using each therapy individually in reducing the deterioration associated with AD [33]. Nevertheless, Moges et al. observed no substantial enhancement in motor function or survival of motor neurons in the anterior horn of the lumbar spinal cord of a transgenic mouse model of familial amyotrophic lateral sclerosis when subjected to a combined laser therapy (810 nm) and riboflavin protocol [99]. Moreover, research has shown that the combination of PBM with exercise has a synergistic impact on mitigating the decline associated with AD [34]. Patients with PD have been found to benefit from combined treatments involving infrared laser and vacuum therapy, as well as molecular hydrogen water treatments. These treatments have been shown to effectively accelerate the relief of disease severity [35, 36].

#### Ischemia

In their study, Lapchak et al. [100] found that the simultaneous use of transcranial near-infrared laser therapy and thrombolytic therapy did not have any impact on the occurrence or size of hemorrhages in a stroke model induced by embolism [100]. Another research conducted demonstrated that the use of red-light emitting diode irradiation in conjunction with bone marrow mesenchymal stem cell transplantation had a synergistic effect on enhancing the movement of stem cells towards damaged primary neurons. This approach also resulted in improved avoidance memory in a rat model of global cerebral ischemia [101]. Moreover, research has shown that the combined use of PBM and Coenzyme Q10 significantly reduced the negative effects of global cerebral ischemia on spatial and episodic memory, excessive production of reactive oxygen species (ROS), neuroinflammation, and impairments in mitochondrial function and biogenesis in a model of aging induced by d-galactose [102]. A clinical trial study demonstrated that the application of both PBM (comprising laser and LED) and static magnetic field treatment resulted in enhanced functional mobility outcomes in individuals who had experienced a stroke [37]. In a similar vein, Ashrafi et al. found that the concurrent use of pulsed LLLT and an extremely low-frequency electromagnetic field reduced the severity of stroke, enhanced cognitive function, alleviated depression, and mitigated the extent of disability in performing daily tasks among individuals who had suffered a stroke [38]. Other studies have shown that the co-administration of PBM with neuromuscular electrical stimulation or a static magnetic field to patients diagnosed with a stroke resulted in optimal improvements in cognitive function, pain relief, and kinematic variables of the hip in both paretic and non-paretic limbs [39, 40].

#### Nerve injury

Studies have shown that using a CO2 laser, along with three distinct suture materials and a bovine albumin protein solder, produces favorable initial histological outcomes and aids in the recovery process at the site of nerve repair [103].

Muniz et al. discovered that the combination of LLLT with natural latex protein reduces the severity of muscle wasting after a sciatic nerve injury (SCI) [108]. In addition, Yang et al. found that the combination of LLLT with mesenchymal stem cells had a more significant impact on the functional recovery of a crushed sciatic nerve compared to using either therapy alone [106]. In addition, the combination of PBM with dexamethasone and simvastatin demonstrated superior efficacy compared to individual therapies in enhancing SCI outcomes [109, 111]. In contrast, certain studies have suggested that the use of combination therapy does not result in a synergistic impact on the recovery from SCI [107, 125].

In their study, Souza et al. found that the concurrent application of transdermal monosialoganglioside (GM1) and laser did not result in any notable impact on the functional and neurological outcomes after SCI in rats [112]. Furthermore, the co-administration of PBM along with chondroitinase ABC or meloxicam has demonstrated enhanced functionality in the identical model [113, 114, 116]. Moreover, there have been reports indicating that the combination of LLLT with either human adipose-derived stem cells or human umbilical cord mesenchymal stem cells has proven to be successful in restoring motor function and promoting the regeneration of nerve fibers in rat models of SCI [115, 117]. A recent randomized clinical trial demonstrated that patients with incomplete spinal cord injury experienced improvements in sensory responses, muscle strength, and muscle contraction one month after receiving a combination of PBM and physiotherapy [41].

### Peripheral Nervous System (PNS)

#### Pain

Prior research has shown that the amalgamation of LLLT with Q10 or oxytocin can elevate thresholds in models of neuropathic pain [121, 122]. Moreover, a randomized controlled clinical trial demonstrated that the combination of LLLT and carbamazepine reduced the intensity of pain in individuals suffering from trigeminal neuralgia [43]. Additionally, a separate study found that the combination of LLLT and Gasserian ganglion block can extend the duration of pain relief and decrease the amount of carbamazepine taken by patients with trigeminal neuralgia after treatment [42].

Studies have shown that the use of PBM in conjunction with exercise or ultrasound therapy can alleviate pain, improve shoulder flexion, elbow extension, and handgrip strength in individuals suffering from lateral epicondylitis [44–46]. Amanat et al. demonstrated the effectiveness of combining laser therapy with pharmaceutical therapy, including tricyclic antidepressants, anxiolytics, muscle relaxants, and carbamazepine, for treating orofacial pain [47]. In addition, Martins et al. demonstrated that long-term combined therapy with PBM and B complex vitamins effectively reduced pain responses [126].

Administering infrared laser therapy in conjunction with exercise or conventional medical interventions (such as naproxen sodium, fluoxetine, and clonazepam) to individuals suffering from myofascial pain syndrome resulted in decreased pain levels and elevated excretion of serotonin metabolites [48, 49, 51]. Furthermore, the simultaneous application of LLLT and physiotherapy resulted in the alleviation of pain and enhancement of the quality of life in individuals suffering from myofascial pain syndrome [50].

Research has shown that the utilization of both infrared laser treatment and physical exercise can effectively alleviate pain in individuals suffering from chronic low back pain [52–54]. Moreover, a clinical trial demonstrated that the use of both hot-pack therapy and two specific wavelengths of low-level laser therapy (850 nm and 650 nm) effectively reduced pain severity and enhanced functionality and range of motion in this particular group [55]. Furthermore, a combined effect on the intensity of pain and the function of the shoulder has been observed when laser therapy is used in conjunction with exercise in individuals diagnosed with subacromial impingement syndrome [60–62].

Kolu et al. discovered that a combination of transcutaneous nerve stimulation (TENS), ultrasound, and exercise yielded superior results compared to high-intensity laser therapy combined with a hot pack and exercise. This combination was found to be more effective in reducing pain and improving functionality in patients with chronic lumbar radiculopathy [65]. Moreover, the concurrent use of PBM with a static magnetic field or active electrical stimulation has demonstrated synergistic effects in alleviating pain intensity in individuals suffering from chronic neck pain [127]. Similarly, the effectiveness of LLLT in combination with ultrasound, exercise, or physiotherapy has been reported to exhibit robust synergistic therapeutic effects in treating shoulder tendonitis [57, 58] and tendinopathy [66, 68, 128].

A combination of laser therapy, chiropractic joint manipulation, ozone therapy, or exercise has been shown to effectively improve cervical flexion, lateral flexion, rotation, and pain disability in patients with cervical facet dysfunction, cervical disc herniation, or spondylosis, when compared to using only one of these treatments [69–72]. Moreover, the application of LLLT and piroxicam has demonstrated favorable outcomes in reducing the intensity of pain in individuals afflicted with temporomandibular joint arthralgia [73].

The utilization of both PBM and manual therapy has been discovered to effectively alleviate pain and jaw impairments, while also enhancing mandibular function in individuals diagnosed with temporomandibular disorders (TMD) [75]. Moreover, multiple studies have utilized a fusion of PBM and ultrasound therapy for TMD treatment. These studies have documented decreases in physical pain and psychological constraints, along with enhancements in quality of life [76–78]. Furthermore, a combination therapy of laser therapy and vacuum therapy has been found to result in pain relief and improvement of TMD joint motion [78]. Combining orofacial myofunctional therapy with PBM has demonstrated favorable results, including decreased pain in patients with TMD [129].

Furthermore, recent findings indicate that individuals suffering from fibromyalgia can experience positive outcomes in terms of decreased pain and enhanced psychological well-being, functional ability, and overall quality of life through the use of adjunct PBM therapy and exercise, or a combination of PBM and ultrasound [80–83]. Furthermore, the combination of laser therapy and ultrasound has been proven to effectively alleviate pain and decrease disability in individuals suffering from osteoarthritis [84].

Gavish et al. found that the efficacy of a combined treatment of LLLT and physiotherapy was superior to physiotherapy alone in managing anterior knee pain in patients. Furthermore, this beneficial effect persisted for a duration of 3 months post-treatment [85].

#### Paresis

The utilization of both LLLT and stellate ganglion block has demonstrated the ability to expedite the process of recuperation from facial paralysis[86]. Yamada et al. found that the use of both LLLT and corticosteroid therapy had a more significant impact on patients with facial palsy in the early stages of recovery compared to using either therapy alone [87]. The combined use of LLLT and facial exercise treatment has shown synergistic effects in patients with facial paralysis. This therapy has been found to enhance functional facial movements and reduce the time required for recovery [88].

#### Neuropathy

Combining LLLT with TENS has been shown to reduce pain scores and median nerve sensory latency, alleviate Phalen and Tinel signs, and enhance functionality in individuals with CTS [89]. Furthermore, a clinical trial validated that the utilization of a combination of a high-power laser (808 nm, 6.5 J/cm2) and TENS alleviated the intensity of pain and enhanced hand functionality in patients with CTS [93]. Dincer et al. found that the concurrent use of LLLT and splinting yielded superior results compared to individual therapies in terms of reducing pain scores and enhancing patient satisfaction [90]. Similarly, Fusakul et al. showed that the utilization of LLLT in conjunction with wrist splinting resulted in reduced pain scores, enhanced hand grip strength and pinch strength, and improved the functional status of individuals with CTS [92].

Nevertheless, a study indicated that the utilization of both kinesiotaping and LLLT in CTS did not exhibit superiority over LLLT alone in the immediate term (3 weeks). Over a period of 12 weeks, the combination of therapies yielded greater improvements in hand grip strength and finger pinch strength outcomes compared to individual therapy [94]. Bartkowiak et al. discovered that the use of LLLT at a wavelength of 830 nm and energy density of 9 J/cm2, along with nerve and tendon gliding exercises, significantly reduced sensory disturbances and pain scores in patients with CTS. Additionally, it improved hand grip strength and functionality. However, they found no additional advantage when comparing it to the combination of ultrasound with nerve and tendon gliding exercises [95].

### Limitation

For this systematic review, some limitations should be highlighted. The lack of details about the parameters in some studies, hindered the possibility of meticulous evaluations. The heterogeneity in included disorders (CNS and PNS) exacerbated the exact focusing on each (made it difficult to focus on each specific disorder). Moreover, there was a limited number of CNS-related interventions in clinical studies. Also, the variation in combined treatment approaches resulted in a lack of uniformity in the data. The stimulation parameters used for performing PBM in the included disorders were not unified. Parameters such as wavelengths, frequency, pulse width, stimulation target, intensity, duration, and unilateral/bilateral treatment differed between the included studies. Due to these limitations, we could only assess the variety of combinations and the effect of key parameters on reported outcomes in the included studies. Another limitation was the moderate quality of the included studies, as assessed using a risk of bias assessment tool. The majority of studies had a pre-post design, were not randomized and blinded.

Despite the limitations of this systematic review, there were also several strengths that are important to mention. We conducted comprehensive research by including both animal and human studies that focused on PBM-combined methodologies which had not been previously mentioned.

Furthermore, we documented all potential combinations that were examined in prior investigations. Moreover, this systematic review covered a wide range of psychological and neurological disorders which is unique. Additionally, we considered multiple scientific databases, providing an overview that is as complete as possible.

## Conclusion

This systematic review clearly demonstrates the therapeutic role of PBM combined therapies, as well as their potential to improve treatment efficacy and reduce side effects across a wide range of central and peripheral neurological disorders. This approach provides numerous research opportunities for studying the synergistic effects of combining PBM with other treatment modalities to optimize neural tissue stimulation by this technique. Also, this review listed the all-possible combinations that studied in previous preclinical and clinical researches. Given the significant heterogeneity in the combined treatment approaches and included disorders, additional studies are required to establish more consistent evidence of efficacy. These studies will provide guidance for the development of well-designed and successful clinical trials.

### Supplementary Information

**Supplementary Material 1.**

## Data Availability

All data generated or analyzed in this work are included in the published version.
