# Correction: Photobiomodulation combination therapy as a new insight in neurological disorders: a comprehensive systematic review

**DOI:** 10.1186/s12883-024-03624-0

**Published:** 2024-04-10

**Authors:** Narmin Farazi, Hanieh Salehi-Pourmehr, Fereshteh Farajdokht, Javad Mahmoudi, Saeed Sadigh-Eteghad

**Affiliations:** 1 Neurosciences Research Center, Tabriz University of Medical Sciences, Tabriz, 5166614756 Iran; 2 Research Center for Evidence-Based Medicine, Iranian EBM Centre: A Joanna Briggs Institute (JBI) Center of Excellence, Tabriz University of Medical Sciences, Tabriz, Iran

**Correction: BMC Neurol 24, 101 (2024)**

10.1186/s12883-024-03593-4

Following publication of the original article [1], the authors reported that the conclusion section was missing in the published article.

The missing conclusion is provided below.

**Conclusion**.

This systematic review clearly demonstrates the therapeutic role of PBM combined therapies, as well as their potential to improve treatment efficacy and reduce side effects across a wide range of central and peripheral neurological disorders. This approach provides numerous research opportunities for studying the synergistic effects of combining PBM with other treatment modalities to optimize neural tissue stimulation by this technique. Also, this review listed the all-possible combinations that studied in previous preclinical and clinical researches. Given the significant heterogeneity in the combined treatment approaches and included disorders, additional studies are required to establish more consistent evidence of efficacy. These studies will provide guidance for the development of well-designed and successful clinical trials.

The original article [1] has been updated.
