# Supplementary material for: Photobiomodulation combination therapy as a new insight in neurological disorders: a comprehensive systematic review
Source: BMC Neurol. 2024 Mar 19;24:101. doi: 10.1186/s12883-024-03593-4 (PMC10949673; doi:10.1186/s12883-024-03593-4)
Supplement: Supplementary file 1 — Supplementary Material 1. [file 12883_2024_3593_MOESM1_ESM.docx]

**PubMed Strategy:**

| Search number | Query | Sort By | Filters | Results |
| --- | --- | --- | --- | --- |
| 4 | (((((((((((("Low-Level Light Therapy"[Mesh]) OR (Low-Level Light Therapy[Text Word])) OR (Low-Level Laser Therapy[Text Word])) OR (LLLT[Text Word])) OR (Photobiomodulation*[Text Word])) OR (PBM[Text Word])) OR (LED[Text Word])) OR (LEDs[Text Word])) OR (Light-emitting diode[Text Word])) OR (light-emitting diodes[Text Word])) OR (light emitting diode*[Text Word])) AND ((((((((((((((("Central Nervous System Diseases"[Mesh]) OR ("Peripheral Nervous System Diseases"[Mesh])) OR ("Ischemia"[Mesh])) OR (Neurolog*[Text Word])) OR (Peripheral Nervous System*[Text Word])) OR (Central Nervous System*[Text Word])) OR (CNS[Text Word])) OR (PNS[Text Word])) OR (Neuropsychiatr*[Text Word])) OR (Neurodegener*[Text Word])) OR (Paresis[Text Word])) OR (Neuropathy[Text Word])) OR (Ischemia*[Text Word])) OR (Nerve Injur*[Text Word])) OR (Pain*[Text Word]))) AND (Combin*[Text Word]) | | | 8,475 |
| 3 | Combin*[Text Word] | | | 2,715,311 |
| 2 | (((((((((((((("Central Nervous System Diseases"[Mesh]) OR ("Peripheral Nervous System Diseases"[Mesh])) OR ("Ischemia"[Mesh])) OR (Neurolog*[Text Word])) OR (Peripheral Nervous System*[Text Word])) OR (Central Nervous System*[Text Word])) OR (CNS[Text Word])) OR (PNS[Text Word])) OR (Neuropsychiatr*[Text Word])) OR (Neurodegener*[Text Word])) OR (Paresis[Text Word])) OR (Neuropathy[Text Word])) OR (Ischemia*[Text Word])) OR (Nerve Injur*[Text Word])) OR (Pain*[Text Word]) | | | 3,745,606 |
| 1 | (((((((((("Low-Level Light Therapy"[Mesh]) OR (Low-Level Light Therapy[Text Word])) OR (Low-Level Laser Therapy[Text Word])) OR (LLLT[Text Word])) OR (Photobiomodulation*[Text Word])) OR (PBM[Text Word])) OR (LED[Text Word])) OR (LEDs[Text Word])) OR (Light-emitting diode[Text Word])) OR (light-emitting diodes[Text Word])) OR (light emitting diode*[Text Word]) | | | 631,366 |
